# Validation of a Questionnaire of Food Education Content on School Catering Websites in Spain

**DOI:** 10.3390/ijerph19063685

**Published:** 2022-03-20

**Authors:** Nuria Rico-Sapena, María Eugenia Galiana-Sánchez, Joaquín Moncho

**Affiliations:** 1 Unidad Técnica Agroalimentaria, Departamento Servicio Territorial de Agricultura de Alicante, Dirección Territorial de Alicante—Consejería de Agricultura, Desarrollo Rural, Emergencia Climática y Transición Ecológica, 03690 Alicante, Spain; rico_nur@gva.es; 2 Balmis Research Group in History of Science, Health Care and Food, NISALdes, University of Alicante, 03009 Alicante, Spain; 3 Research Unit for the Analysis of Mortality and Health Statistics, Department of Community Nursing, Preventive Medicine, Public Health and History of Science, University of Alicante, 03080 Alicante, Spain; joaquin.moncho@ua.es

**Keywords:** validation questionnaire, websites, catering, health promotion, food education

## Abstract

The aim of this study was to design and validate a questionnaire on quality and food education on catering company websites. For the validation of the questionnaire, its reliability, feasibility and content validity characteristics were determined. For content validity, a panel of experts was used and the overall and item-wise Content Validity Index (CVI) of the experts’ responses was calculated. Reliability was determined by the inter-judge agreement method with the analysis of 30 websites using the intraclass correlation coefficient (ICC) and the Bland and Altman plot. Adequate content validity was verified by obtaining a very high CVI (above 0.80) in the analysis of the expert panel’s responses and excellent inter-observer agreement with a very high global ICC (above 0.90) value in the determination of test-retest reliability. The questionnaire is a valid instrument for the evaluation of the quality of school catering websites and their content in food education, as it has good feasibility, high content validity and excellent reliability.

## 1. Introduction

Children’s dietary habits during childhood are linked to risk of developing chronic conditions and food consumption patterns in adulthood [1,2,3,4,5,6]. Unhealthy food and lack of physical activity have contributed to increasing the prevalence of overweightness and obesity in children and adolescents [3,7,8,9,10,11,12,13,14,15,16]. Obesity is considered by the WHO as the disease of the 21st century due to the dimensions it acquires, and the impact on health, quality of life and health spending [10,11,17]. Therefore, obesity is a complex public health issue that requires large-scale and population-based solutions [18].

Schools are the places where children spend most of their time and have access to a significant part of the food they eat [1,18]; therefore, they are key settings for the prevention of obesity [18], health promotion [1] and the consolidation of correct eating habits [19]. School canteens, in the current social context, are essential elements in child nutrition and in the achievement of public health objectives [2,7,19,20,21,22].

The school canteen catering service plays an important food, nutritional and educational role by providing a varied and nutritionally balanced diet, and by being a powerful tool for food and nutrition education and improving healthy eating habits [6,20,22,23,24,25].

Catering companies use the Internet environment to present their services and activities. Their websites allow them to show different activities and educational content on food, nutrition and healthy eating habits. It is important that this educational contribution is adequate, both in terms of content and the quality of the website itself, so that this information translates into health education for users [26,27,28].

The increase of information on the Internet favours the development of multiple websites whose quality and content characteristics are highly variable, making it increasingly difficult to select information and ensure that the results are of high quality [28,29,30,31]. For this reason, the evaluation of websites has become essential to be able to contrast this type of information [26,29,30]. 

Questionnaires are the instrument that has been used in numerous previous studies [26,27,28,31,32,33,34,35,36,37,38,39,40,41] for website evaluation. However, for an instrument to be useful, it must be valid, reliable, easy to apply and usable in any environment [42,43,44]. Evaluating the psychometric properties of an instrument is an essential criterion for determining the quality of its measurement [45], being a continuous and dynamic process that becomes more consistent the more psychometric properties are measured with different populations, subjects and in different cultural contexts. It is a complex process involving the assessment of feasibility, reliability, validity and sensitivity to change [45,46,47,48,49].

Several previous studies assessed the quality of web page features but did not analyse the educational content or the quality of the content of the web pages [26,27,28,31,32,33,34,35,36,37,38,39,40,41], which would require appropriate instruments to do so. In a previous phase of the present study, a first version of the EDALCAT questionnaire was designed, and its feasibility was assessed by means of a pilot test. This pilot test was carried out in order to assess the appropriate wording of the items and to adjust the quality criteria of the website. The aspects that were evaluated were: the time required to complete it, the simplicity of the format, the interest, brevity and clarity of the questions, as well as the recording, coding and interpretation of the results. It was also useful to carry out a first approximation of the food education content on the websites analysed [45,46,50].

The aim of this study was to assess the content validity and reliability of the questionnaire and to obtain the final design of the Questionnaire on evaluation in catering education (EDALCAT).

## 2. Materials and Methods

The EDALCAT questionnaire is a self-made checklist questionnaire, which initially included thirty-five items, with dichotomous questions (yes or no) for evaluation on quality and food education content and quality on catering company websites [50].

EDALCAT is composed of two parts, the first a block of predictors of website quality, divided into three subsections: Reliability, Design and Navigation. The items included in these sections were obtained by consulting the literature on the subject [27,28,30,32,33,34,35,36,37,51,52,53,54], and the recommendations of codes of conduct and certifications such as the American Medical Association (AMA), the e-Health Code of Ethics of the Internet Heath Coalition, the Health Summit Working Group (Summit), the e-Europe 2002 code, Accredited Medical Web (WMA), and the Health on the Net Foundation (HON Code), among others.

The second block of the EDALCAT incorporates two subsections relating, on the one hand, to the specific content of food education, and on the other hand, to the possible educational activities that caterers develop in schools. To determine the items in this section, the literature on food and nutrition education was consulted [20,55,56,57].

For the validation of the questionnaire, its reliability and content validity characteristics were determined as the feasibility was determined in a previous experiment consisting of a pilot study with ten selected websites [50].

To determine the content validity of the questionnaire, a panel of 14 experts was set up, taking into consideration their professional and research experience, and their recognised prestige in the field of community nutrition, public health, and informatics, which allowed for a comprehensive analysis of the problem under study. The experts were formally invited to participate in the project by e-mail. A computer application was developed with a questionnaire to assess the suitability of the items of the EDALCAT questionnaire for online completion. They were asked to make an overall assessment of the questionnaire and of the suitability of each of the items. They analysed the content of the questions in terms of comprehensibility, format and applicability, and assessed whether the items were relevant and representative of the domain they were intended to measure. The items were scored on a Likert scale from 1 to 5 (1 = Strongly disagree, 2 = Disagree, 3 = Not sure, 4 = Agree and 5 = Strongly agree). They were also allowed to make any suggestions about the questionnaire.

The statistical analysis was carried out by first obtaining the means and standard deviations of the scores given by the experts, as well as the Content Validity Index (CVI). This index was calculated by taking two different cut-off points. The first cut-off point was more demanding (4 or more) and the second less restrictive (3 or more), in line with those used in other studies [58,59,60,61]. The Inter-rater Agreement Method or inter-observer reliability was used to determine test-retest reliability. Two new raters who had not been members of the expert panel and with experience in the field of food and nutrition were selected to evaluate 30 selected websites with the EDALCAT. One of these new raters also had extensive experience in website design and evaluation and was considered the gold standard [46]. The intraclass correlation coefficient (ICC) was calculated for the total score and for each of the five sections of the questionnaire. In addition, scatter plots and Bland and Altman plot were constructed for the total scores obtained from the questionnaire by both raters.

Statistical analysis was performed using the statistical package PASW IMB SPSS Statistics 28 Microsoft Co., New York, NY, USA.

## 3. Results

**Figure 1:**
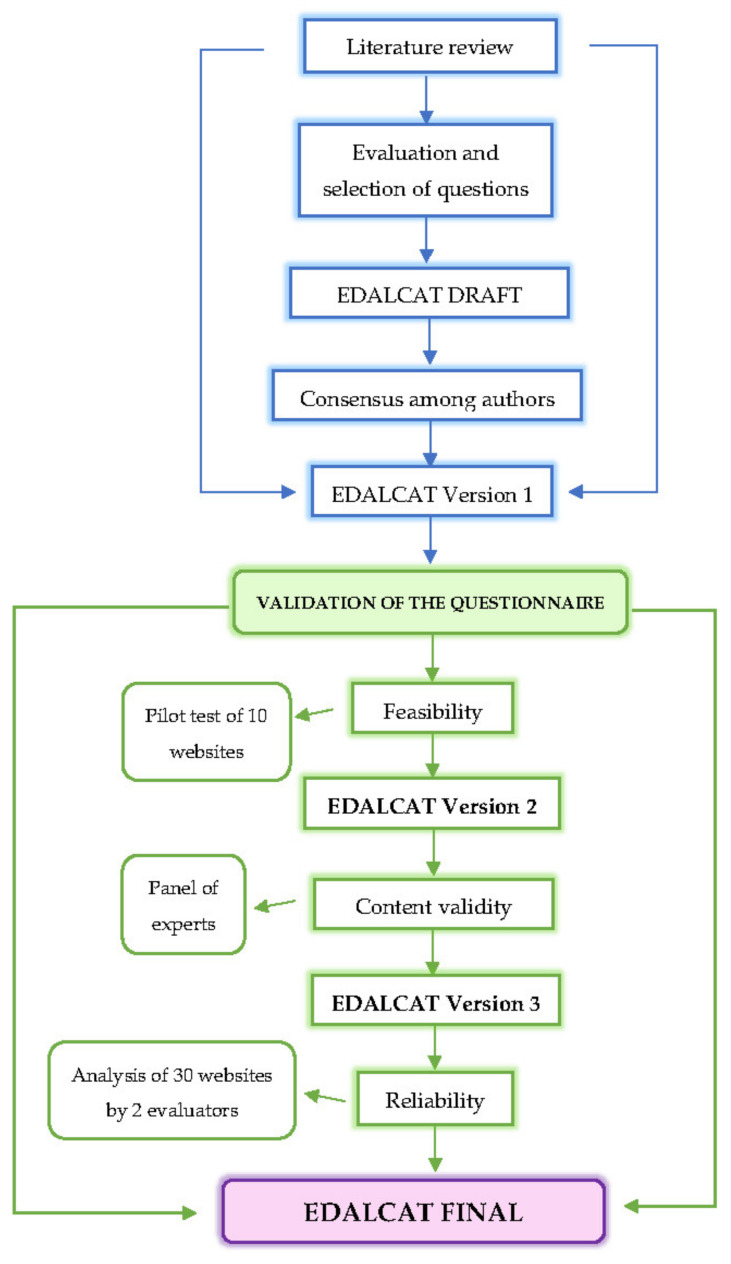
Design and validation phases of the EDALCAT questionnaire.

The process of creating and validating the questionnaire with its different phases is shown in the flowchart in Figure 1.

Following the feasibility assessment [50], a second version of the EDALCAT questionnaire was obtained, which included the numbering of the different items to facilitate their referencing and the modification of the wording of two of them. Specifically, items 12 and 14, which became “Is the information contained on the website in more than one language?” and “Does it have an adequate browsing speed?”, respectively.

### 3.1. Content Validity

The content validity results from the expert panel can be seen in Table 1. All questions scored a mean of 4 points or more, except questions 6 and 34.

The CVI^(1)^ indicates the percentage of scores between 4 and 5 that each item received from the experts. The items with the lowest CVI^(1)^ were 33 and 34, although it should be noted that none of the items scored less than 3.

The CVI^(2)^ indicates the percentage of scores between 3 and 5 for each item. The CVI^(2)^ was 100% for all items except for question number six, which scored 71.40%, due to the fact that only one assessor scored 2 points.

After analysing the experts’ responses to the questionnaire and their suggestions, various modifications were made to several of the items in the questionnaire, as well as to the definitions of the variables.

In the Reliability section, question three was modified, replacing “Is the information up to date?” with “Does the web page indicate when it is up to date?”. In addition, the definition of the variable update was changed to “Date of last modification of the website”.

In the Design section, it was not considered necessary to modify any item.

In the Navigation section, items 15 and 17 were modified to read, “Is it easy to find the contents of the website?” and “Are there any recommended links?”, respectively. The question “Does the website have access to social networks?” was added. In addition, the definitions of the variables corresponding to questions 14 (speed) and 16 (downloads) were modified to read, “Adequate browsing speed, waiting time to load the website less than 5 sec.” and “Existence of any material to download, photos, documents, etc.”, respectively.

In the Content section, questions 22 and 24 were combined, resulting in the question “Is the information contained on the site factual and nutritionally adequate?”. In question 23, the word “unambiguous” was deleted, and the wording was changed to “Is the information comprehensible?”. Finally, question 30 was split into two questions, “Do you provide nutritional information on the school menu?” and “Do you provide recommendations for supplementation of the school menu?”.

### 3.2. Reliability

Table 2 shows the results of the reliability analysis of the questionnaire through inter-observer agreement, where an overall ICC value of 0.996 can be observed. Furthermore, the ICC calculated in each of the dimensions of the questionnaire was higher than 0.99 except in the Design and Navigation sections, where ICC values of 0.871 and 0.972, respectively, were obtained.

**Figure 2:**
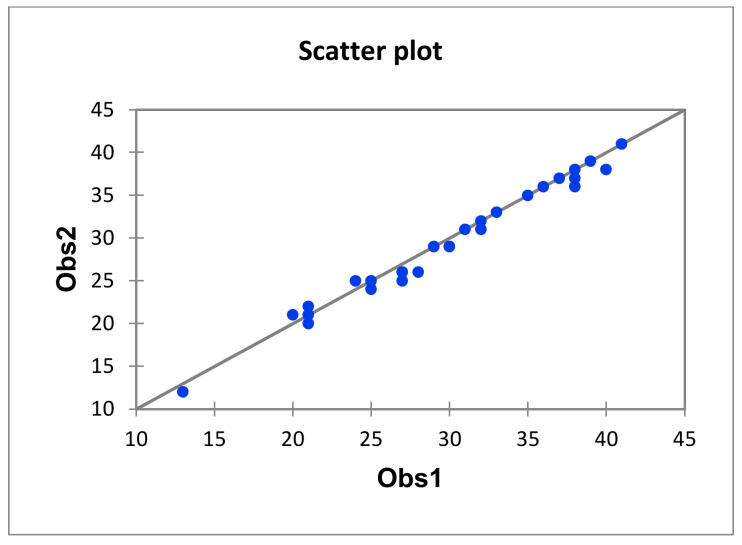
Inter-observer agreement scatterplot.

Figure 2 shows the scatter diagram of the total scores of the web pages awarded by each of the evaluators. It can be seen that the points are located around the bisector.

**Figure 3:**
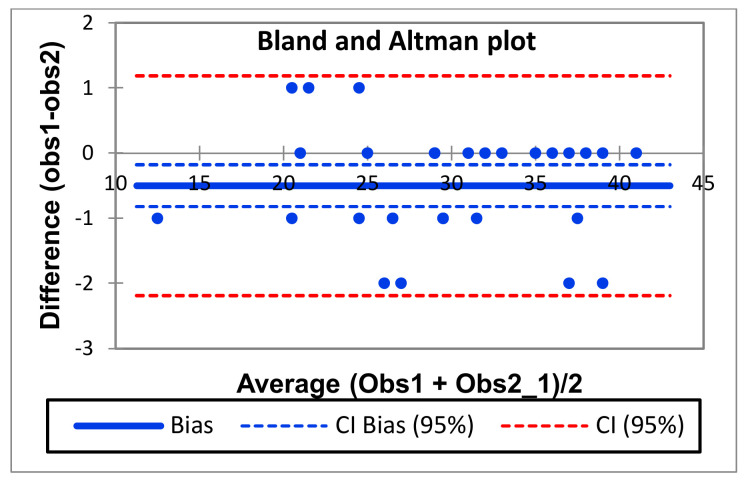
Bland-Altman Graph.

The Bland–Altman plot in Figure 3 represents, on the ordinate axis, the difference between the scores of the two observers, and on the abscissa axis, the mean of the scores obtained for each of the web pages. It can be seen that the difference remains approximately constant and within the 95% confidence interval. A small bias is detected whereby observer one would be scoring, on average, 0.5 points lower than observer two.

With these results, the adequacy of the items of the questionnaire was checked, and it was not necessary to modify any of them; it was only considered necessary to number the educational activities.

Finally, in the definition of variables, some adjustments were made to improve the wording and to clarify them, such as the definition of “Language”, or “Food-nutrition website”. The result was the final EDALCAT questionnaire (Table 3) together with the definition of variables (Table 4).

## 4. Discussion

EDALCAT presented optimal psychometric properties to be used as an instrument to assess the quality of catering websites and their food education content.

The experts’ scores for the different items of the questionnaire in their content validity were very high in all cases, with average values above 4 out of 5 points in all of them, except in the case of “Search engine” and “Examples” with scores of 3.86 points. To assess the content validity of EDALCAT, the Content Validity Index (CVI) was chosen because it is easy to calculate and interpret, because it focuses attention on the experts’ agreement on the relevance of the items and because it provides information both on a global scale and for each item [58,59,60,61,62,63]. The CVI has been chosen among other indices in numerous studies in a variety of settings [58,59,60,61,62,63,64,65,66], and its benefits over other indices stand out, as described in the literature [63]. The overall score was 0.87 for the CVI with cut-off point 4 and 0.99 for cut-off point 3, indicating very high content validity, and higher than that obtained in other studies. The study on the PES-NWI questionnaire [58] in the nursing setting obtained an overall average CVI of 0.85 from the 14 versions of the instrument, ranging from 0.60 to 0.98. It should be noted that a lower cut-off point of 3 (on a Likert scale of 1 to 4), and therefore less demanding, was used. Another study [66] on the validation of an instrument for the analysis of sports training in volleyball obtained a CVI of 0.91, while in the case of the Food Safety Systems Functionality Assessment (FSIA) questionnaire to measure the functionality, compliance and operability of national food safety systems, this index was 0.78 [59]. As a general rule, a CVI above 0.80 defines the item set as adequate [58,60,61,62,64,67]. However, it should be taken into account that the degree of agreement on an item also adjusts to the number of experts participating in the assessment and may be lower with a higher number of experts in the panel [60,61,62,63,64]. Considering that our expert panel consisted of 14 members, the CVI obtained can be considered very high for the EDALCAT questionnaire.

When analysing the experts’ responses and suggestions to the questionnaire, several modifications were made to items and variable definitions in order to clarify and facilitate the understanding of the questions in the questionnaire. The items of the variables “Updating”, “Ease”, “Links”, “Speed”, “Downloads” and “Comprehensibility” were modified, and the definition of these variables, such as “Updating”, was changed to “Date of last modification of the website”, as this was considered a more objective indicator of the age of the website. The items of the variables “Objectivity” and “Accuracy” were also reformulated and merged, and the item “Nutritional information and recommendations” was separated, giving rise to two items “Nutritional information” and “Recommendations”, as these are different concepts that could be misleading. Finally, the item “Social networks” was added, as it was considered appropriate to address new technologies and their current frequent use. Although there are other items that scored less well, they were considered appropriate because they are quality standards used as indicators of website quality.

The inter-observer agreement for assessing the reliability of the EDALCAT questionnaire was determined simultaneously by the two assessors. In this way, possible updates of the web pages, whose contents may vary over time, were prevented from influencing the results. Many other studies [44,45,68,69,70,71,72,73] in different fields have also chosen this method to determine the reliability of instruments.

The intraclass correlation coefficients (ICC), to determine the inter-observer reliability of the EDALCAT questionnaire, showed very high values above 0.970 in all dimensions except for the Design dimension, where the ICC value was 0.871. This last value, however, is very acceptable (above 0.75) [74], and its lower magnitude could be explained by its greater subjectivity compared to the rest of the dimensions. The overall ICC was very high and stood at 0.996 (above 0.90) [74], so it is considered to have excellent inter-observer reliability. Several studies obtained similar results, such as the evaluation of the Spanish adaptation of the “Induction Compliance Checklist” [73], which assesses the child’s behaviour during anaesthetic induction, and showed an ICC of 0.956. For its part, the IMS-Es, which assesses the mobility of critical patients in the ICU [75], obtained concordant values between nurses (0.987 ICC) and between nurse-physiotherapists (0.963 ICC). In addition, in the case of the reliability of a questionnaire on modes, time and distance of travel in university students [76], results were obtained that confirm its high reliability, with an ICC of 0.96 for the distance to and from school and 0.95 for the distance to and from university. However, other studies obtained significantly lower values, as in the study by Llorens-Ivorra et al. 2017 [71], which evaluated a questionnaire on food balance in school menus, where a value of 0.868 was reached for the overall ICC of food groups. Similarly, in the Spanish translation and validation of the Paediatric Quality of Life Inventory scale [72] on quality of life perceived by parents of children with neuromuscular diseases, an overall ICC of 0.90 was obtained. On the other hand, in studies on the evaluation of the questionnaire to measure knowledge about medication in patients [44] and in the evaluation of websites on information in the primary care setting [45], overall ICC values of 0.75 and 0.73, respectively, were obtained, which are significantly lower than those obtained in our study.

The reliability study was completed with a scatter plot and the Bland–Altman graph constructed from the total scores of the web pages given by each of the two evaluators. This method is used in some studies [69,71,72] to provide additional and alternative information, in a very visual and simple way, about the reliability of questionnaires. Both graphs showed high inter-observer agreement. Although a bias was detected whereby one of the observers was giving lower scores than the other, its magnitude was very small in relation to the overall score, at 0.5 points. On the other hand, the 95% confidence interval for the difference between the scores contained all observations and showed an acceptably low range of no more than two points difference between its upper and lower limit, regardless of the magnitude of the mean score. The results of such high inter-rater agreement are evidence of reliability in the EDALCAT questionnaire.

When checking the adequacy of the questionnaire items, it was not necessary to modify any of them. Only some editorial adjustments were made to the definitions of the variables in the questionnaire that could have a certain degree of subjectivity in their interpretation, such as “Language” or “Food-nutrition website”.

The limitations of the present study include, firstly, the dynamic and constantly updated nature of the information on the web pages, which means that the results have a temporal validity. In our study, we tried to minimise this circumstance by carrying out the evaluation of the experts on a simultaneous basis. On the other hand, the information contained in the web pages is of great interest, but all virtual information should be contrasted with information in situ, in order to verify its veracity and the scope of the educational activities carried out. Therefore, future research could focus on analysing the educational activities carried out in school canteens by catering companies, in collaboration with schools and educators, to check their involvement in practice and their impact on children.

The items relating to educational activities in the EDALCAT questionnaire could be useful for future evaluations of the educational activities carried out in school canteens, although it would be necessary to have additional data such as those relating to objectives, resources, content, methodology, action and evaluation.

The current development of the Internet has changed the way we relate to each other and obtain information, a fact that catering companies can take advantage of in order to offer services to users according to their needs. In addition, the number of children using school canteens and parents who are more aware of the importance of a proper diet for their children’s health is increasing every day. Catering websites are a good tool to provide quality information on food education, nutrition and health, provided that the information is endorsed by professionals and of high-quality standards.

Finally, the results of our study could guide the direct educational interventions of catering companies in school canteens and give greater relevance to food and nutrition education. The EDALCAT questionnaire, in this case, could be useful for the production of higher quality websites in relation to food education content.

## 5. Conclusions

This study developed and validated an EDALCAT questionnaire for the evaluation of catering websites, not only to measure the quality of the websites but also to assess their content in educational activities on food in school canteens.

The EDALCAT questionnaire developed in this research is a valid instrument for the evaluation of school catering websites, as the analysis of its psychometric properties showed it to have good feasibility, high content validity and excellent reliability. It is also an instrument with a possible international application and in areas other than this study.

This questionnaire can help catering companies to give more relevance to food and nutrition education through educational interventions and improvements in the activities and services provided in school canteens.

The results of this research could be considered as an opportunity for improvement, inviting managers of catering companies to implement better practice in the development of their websites.

## Figures and Tables

**Table 1:** Average of experts’ scores.

Items of the Version 2 Questionnaire by Sections **Reliability**	**Mean**	**Stdev**	**CVI (1)**	**CVI (2)**
1. Is the author(s) identified on the website?	4.71	0.49	100.00	100.00
2. Is a contact address for the catering company provided?	4.86	0.38	100.00	100.00
3. Is the information up to date?	4.29	0.95	71.40	100.00
4. Is the protection of personal data and privacy specified?	4.29	0.76	85.70	100.00
5. Does the website indicate the quality certificates of the catering company?	4.14	0.90	71.40	100.00
6. Does it contain an internal search engine for the information contained on the website?	3.86	1.35	71.40	71.40
**Design**				
7. Does the website have an attractive and original graphic and multimedia design?	4.29	0.76	85.70	100.00
8. Is the website properly structured and organised?	4.86	0.38	100.00	100.00
9. Does it include a site map?	4.43	0.79	85.70	100.00
10. Does it have a clear and appropriate language for the user?	4.71	0.49	100.00	100.00
11. Is the font size and colour contrast appropriate?	4.14	0.69	85.70	100.00
12. Is the information contained on the website in more than one language?	4.00	0.82	71.40	100.00
**Navigation**				
13. Is the website accessible and easy to navigate?	4.86	0.38	100.00	100.00
14. Does it have an adequate browsing speed?	4.14	0.90	71.40	100.00
15. Is it easy to find content and search the website?	4.71	0.76	100.00	100.00
16. Does it have downloadable material?	4.29	0.95	71.40	100.00
17. Are there recommended links and are they up to date?	4.29	0.76	85.70	100.00
18. Does it have online help?	4.29	0.95	71.40	100.00
19. Is the site free of advertising?	4.86	0.38	100.00	100.00
**Content**				
20. Is the objectives or mission of the caterer reflected on the website?	4.29	0.95	71.40	100.00
21. Does it have a section on food, nutrition or dietetics?	4.43	0.79	85.70	100.00
22. Is the information on the website objective?	4.86	0.38	100.00	100.00
23. Is the information comprehensible and unambiguous?	4.86	0.38	100.00	100.00
24. Is the information nutritionally correct and adequate?	4.57	0.79	85.70	100.00
25. Is the content endorsed by competent professionals?	4.57	0.53	100.00	100.00
26. Does it relate nutrition to health?	4.57	0.53	100.00	100.00
27. Is the importance of physical exercise indicated?	4.29	0.76	85.70	100.00
28. Does the caterer participate in food and nutrition education programmes?	4.43	0.79	85.70	100.00
29. Are special menus provided for students with specific therapeutic or cultural needs?	4.57	0.53	100.00	100.00
30. Do you provide nutritional information and recommendations for supplementation of the school menu?	4.57	0.53	100.00	100.00
31. Do you have private access to school menus for parents?	4.71	0.49	100.00	100.00
32. Is the role of educators and their training indicated?	4.29	0.76	85.70	100.00
33. Does the school have a separate website specifically for food and nutrition?	4.00	1.00	57.10	100.00
34. Does the website cite examples or show real cases?	3.86	0.90	57.10	100.00
**Educational Activities**				
35. Are there any activities?	4.57	0.79	85.70	100.00
36. Informative talks	4.43	0.79	85.70	100.00
37. Gastronomic days	4.57	0.79	85.70	100.00
38. Cooking workshops	4.57	0.79	85.70	100.00
39. Healthy cooking recipes	4.71	0.49	100.00	100.00
40. Other	4.43	0.79	85.70	100.00
**Global**	4.44	0.71	87.00	99.00

^(1)^ Content Validity Index based on % of scores 4 or higher. ^(2)^ Content Validity Index based on % of scores 3 or higher.

**Table 2:** Average observer scores and intraclass correlation coefficient.

	OBSERVER 1		OBSERVER 2		ICC
	Mean	Stdev	Mean	Stdev	
Reliability	4.57	1.073	4.60	0.968	0.992
Design	4.73	1.048	4.50	1.106	0.871
Navigation	6.53	1.279	6.27	1.437	0.972
Content	10.20	3.708	10.23	3.626	0.999
Educational Activities	3.87	2.030	3.80	2.041	0.996
Total	29.90	6.880	29.40	6.806	0.996

**Table 3:** Validated EDALCAT questionnaire, for the evaluation of catering websites.

Catering Website			
Predictors of quality			
**Reliability**		YES	NO
1. Is the author(s) identified on the website? 2. Is a contact address of the catering company provided? 3. Does the website indicate when it was updated? 4. Is the protection of personal data and privacy specified? 5. Does the website indicate the quality certificates of the catering company? 6. Does it contain an internal search engine for the information contained on the website?			
**Design**		YES	NO
7. Does the website have an attractive and original graphic and multimedia design? 8. Is the website properly structured and organised? 9. Does it include a site map? 10. Does it have a clear and appropriate language for the user? 11. Is the font size and contrasting colour appropriate? 12. Is the information contained on the website in more than one language?			
**Navigation**		YES	NO
13. Is the website accessible and easy to navigate? 14. Does it have an adequate browsing speed? 15. Is it easy to find content on the website? 16. Does it have downloadable material? 17. Are there any recommended links? 18. Does the website have access to social networking sites? 19. Does it have online help? 20. Is the website free of advertising?			
Specific food education content			
**Contents**		YES	NO
21. Does the website reflect the objectives or mission of the caterer? 22. Does it have a section on food, nutrition or dietetics? 23. Is the information contained on the website objective and nutritionally adequate? 24. Is the information comprehensible? 25. Is the content endorsed by competent professionals? 26. Is food related to health? 27. Is the importance of physical exercise indicated? 28. Does the caterer participate in educational programmes on food and nutrition? 29. Are special menus provided for pupils with specific dietary or cultural needs? 30. Do you provide nutritional information on the school menu? 31. Do you provide recommendations for supplementation of the school menu? 32. Do you have private access for parents to the school menu? 33. Is the role of educators and their training indicated? 34. Does the school have a separate website specifically for food and nutrition? 35. Does the website cite examples or show real cases?			
**Educational activities**		YES	NO
36. Do activities take place?			
What kind of activities does the caterer carry out?	37. Informative talks 38. Gastronomic days 39. Cooking workshops 40. Healthy cooking recipes 41. Other		

**Table 4:** Definition of final variables of the validated EDALCAT questionnaire.

Variables	Definition
Predictors of quality	
**Reliability**	
Authorship	Person/people or company responsible for the content of the website.
Contact address	Address, e-mail address, telephone number, etc.
Updating	Date of last modification of the website.
Privacy	Data protection and user privacy policy.
Certification	Catering quality, food safety, environmental, etc., certifications.
Search	Mechanism for searching, consulting and locating the contents of the website.
**Design**	
Graphic design	Graphic and multimedia design of the website.
Structure	Structure and organisation of the website.
Site map	Site map to facilitate the search of contents.
Legibility	Language and expression of the contents of the website.
Form and colour	Font size and contrasting colour on the website.
Language	Possibility of reading the website in more than one language.
**Navigation**	
Accessibility	Easy access to the information contained in the pages without limitation.
Speed	Adequate browsing speed, web page loading time of less than 5 s. Adequate speed is considered adequate if it has a waiting time of less than 5 s.
Ease	Ease or not of finding content on the website.
Downloads	Existence of any kind of downloadable material, photos, documents, etc.
Links	Existence of links on the website that work correctly.
Social Networking	Existence of hyperlinks to access social networks, Facebook, Twitter, …
Help	Online help for queries from the website, with chat, telephone, …
Advertising	No advertising or promotion of brands or collaborating companies.
Specific Contents	
**Content**	
Purpose	Objective or mission of the services provided by the catering service.
Food and nutrition section	Existence of a specific section on food, nutrition or dietetics on the website.
Objectivity	Information expressed in an objective and nutritionally adequate manner.
Understandable	Information expressed in an understandable and unambiguous way.
Endorsed	Information endorsed by professional and academic persons or institutions.
Health	Content relates nutrition to health effects.
Physical exercise	The content expresses the importance of physical exercise as part of healthy habits.
Collaboration	The catering service collaborates with an established educational programme or has one of its own.
Special menus	The catering service provides special menus for pupils with diabetes, food allergies, coeliac disease, religious or cultural beliefs.
Nutritional information	Nutritional information of the school menu indicating calorie, protein, fat and carbohydrate intake.
Recommendations	Recommendations for supplementing the school menu on the most appropriate food intake for the rest of the day.
Private access	Private access to the school’s monthly menus from the website.
Monitors	Indication of the roles of educators or monitors and their training.
Food-nutrition website	Separate food and nutrition specific webpage separate from the catering website.
Examples	Citation of examples or sample real cases of catering services.
**Educational Activities**	
Activities	Activities aimed at educating about food, nutrition, etc., are carried out.
Talks	Informative talks on food, nutrition and dietetics.
Conferences	Gastronomic days on regions or countries, themes, festivities, etc.
Workshops	Cooking workshops, where children can learn about cooking, food, etc.
Reception	Healthy cooking recipes.
Other	Carrying out activities other than those mentioned above.

## Data Availability

Not applicable.
